# The anaerobic fungus *Neocallimastix californiae* shifts metabolism and produces melanin in response to lignin-derived aromatic compounds

**DOI:** 10.1186/s13068-025-02696-5

**Published:** 2025-08-29

**Authors:** Thomas S. Lankiewicz, Bashar Amer, Edward E. K. Baidoo, Patrick A. Leggieri, Michelle A. O’Malley

**Affiliations:** 1 Department of Chemical Engineering, University of California Santa Barbara, Santa Barbara, CA USA; 2 Department of Ecology, Evolution, and Marine Biology, University of California Santa Barbara, Santa Barbara, CA USA; 3 Joint BioEnergy Institute, Lawrence Berkeley National Laboratory, Berkeley, CA USA; 4 Department of Bioengineering, University of California Santa Barbara, Santa Barbara, CA USA

**Keywords:** Anaerobic fungi, Lignin, Aromatic, Melanin, Metabolism

## Abstract

**Background:**

Biological deconstruction of lignocellulose for sustainable chemical production offers an opportunity to harness evolutionarily specialized enzymes and organisms for industrial bioprocessing. While hydrolysis of cellulose and hemicellulose by CAZymes yields fermentable sugars, ligninolysis releases a heterogeneous mix of aromatic compounds that likely play a crucial role in shaping microbial communities and microbial metabolism. Here, we interrogated the metabolomic and transcriptomic response of a lignocellulolytic anaerobic fungus, *Neocallimastix californiae*, to a heterogeneous mixture of aromatic compounds derived from lignin.

**Results:**

Through exposing the fungus to both a concentration it might experience in its native environment and an elevated concentration of alkaline lignin, we observe that *N. californiae* transforms vanillin and that supplying alkaline lignin at 0.125 g/L, alongside cellulose, enhances the growth and polysaccharide-degrading activity of *N. californiae.* Altogether, our results further suggest that vanillin consumption, increased polymer-degrading activity, increased metabolic activity, and transcriptomic remodeling of amino acid synthesis genes all coincide with increased melanin production by fungal cells. These observations challenge previous notions that aromatics from lignocellulose only inhibit the growth and polymer deconstruction capabilities of the biomass-degrading anaerobic fungi (Neocallimastigomycetes).

**Conclusions:**

This study demonstrates that anaerobic fungi have a complex relationship with aromatic chemicals derived from lignin and hemicellulose and shift their metabolism in response to the addition of lignocellulose-derived aromatics to their growth medium. Further, as no known pathways for the biochemical transformation of aromatics were detected in these organisms despite observed transcriptome remodeling in the presence of aromatics, we suggest they might encode novel biochemical routes for scavenging amino acid building blocks from aromatic monomers derived from hemicellulose side chains and lignin.

**Supplementary Information:**

The online version contains supplementary material available at 10.1186/s13068-025-02696-5.

## Background

Innovation in producing sustainable, carbon-neutral chemicals is equally vital to efforts for decarbonizing energy and transportation. The deployment of synthetic biology to aid in green manufacturing of consumer goods and chemicals is one promising avenue of biotechnological innovation [1]. Biology has evolved to efficiently produce diverse and valuable chemicals from renewable carbon sources such as lignocellulose, the composite polymer constituting cell walls in higher plants [2–4]. Herbivore microbiomes, which rapidly and efficiently extract nutrition from lignocellulose, offer an opportunity to develop lignocellulolytic biotechnologies inspired by millions of years of evolution [5, 6]. By learning from these highly specialized and complex biological systems, it may be possible to borrow enzymes and chemical processes from them, develop scalable and carbon-neutral solutions, and then deploy these bioinspired processes at industrial scale [2, 7].

Anaerobic microorganisms from herbivore guts are well-documented degraders of plant biomass, and among lignocellulolytic anaerobes, fungi of the Class Neocallimastigomycetes are some of the most proficient lignocellulose degraders. These anaerobic fungi are difficult to transform [8, 9], have highly complex genomes [10, 11], propagate through a non-model lifecycle [12, 13], and encode more carbohydrate-active enzymes (CAZymes) than any other organism [14–16]. Anaerobic fungi are already sources of lignocellulose-degrading enzymes and sugar transporters, and most recently attracted attention for their newly discovered ability to break β-aryl-ether (β-O-4) and dibenzodioxin (β-5) bonds in the recalcitrant, aromatic polymer lignin [17, 18]. Despite the observation of apparent lignin deconstruction by Neocallimastigomycetes, only minimal information about how anaerobic fungi sense and subsequently interact with lignin and lignin-derived aromatics, as well as the aromatics present in hemicellulose sidechains, has been documented. Continued descriptions of anaerobic fungal metabolism and sensing are essential to maximizing Neocallimastigomycetes’ contributions to biotechnology [7, 19].

Current models of anaerobic gut fungal metabolism focus on the fate of hexose and pentose sugars, and less information is available on whether Neocallimastigomycetes can sense and use non-sugar carbon sources, like the aromatic byproducts of lignocellulose degradation [20–22]. Anaerobic bacteria that evolved with lignocellulose have complex relationships with aromatic chemicals derived from lignin and hemicellulose [23, 24]. However, anaerobic fungi are not known to encode any homologs to canonical, prokaryotic pathways for anaerobically metabolizing aromatic compounds [15, 25]. Current models of anaerobic fungal metabolism predict mixed acid fermentation pathways consuming sugars and subsequently accumulating hydrogen gas and short-chain fatty acids [20, 26]. Anaerobic fungi also contain enigmatic hydrogenosomes suspected of aiding in energy conservation, but their current benefit to the organisms is unclear [26–29]. Interrogating if and how anaerobic fungi shift their metabolism in response to amendments of aromatic compounds is a first step toward understanding how these organisms’ interactions and biochemical relationships with aromatics differ from those of anaerobic bacteria, as well as the extent to which they can be grown on such compounds.

Most knowledge concerning interactions of fungi with lignin surrounds the action of laccases and peroxidases, which are encoded by aerobic fungi [3, 30], but recently published results suggest anaerobic fungi deconstruct lignin [17]. The means by which anaerobic fungi sense and interact with aromatic constituents of lignin and hemicellulose has received more limited attention [31]. Anaerobic gut fungi transcriptionally respond to various stimuli, but most documented responses are related to carbohydrate metabolism [16, 17, 32, 33]. Differential expression experiments have illuminated patterns of catabolite repression in response to various lignocellulose and purified carbohydrates [17, 18, 33, 34]. Interactions between anaerobic fungi and prokaryotic co-cultivars have also been investigated, demonstrating CAZyme regulation mediated by cross-feeding with a methanogen [21] and also an antagonistic relationship with a bacterium [35]. Anaerobic fungi additionally transcriptionally respond to physical cues, such as stirring, which has implications for these organisms finding and attaching to their carbon sources [21]. One common perspective on Neocallimastigomycetes’ relationship with lignin is that lignin is an inhibitory polymer that prevents access to carbohydrates and inhibits fungal CAZymes. However, transcriptional response to aromatics beyond increased CAZyme expression levels would be indicative of a more complex relationship than simple inhibition and inactivation of extracellular enzymes.

In fungal biology, aromatic amino acids are essential building blocks for melanin. Melanin is a disordered aromatic polymer that is responsible for protection from oxidation from ultraviolet light in the fruiting bodies of higher fungi and the skin of animals [36–38]. Melanin is produced from the polymerization of aromatic amino acids, most commonly via sequential conversion of tyrosine to phenylamine, L-DOPA, and, finally, melanin. Melanin production is a feature of the fungal kingdom, and it has been previously suggested, but not confirmed, that Neocallimastigomycetes produce melanin analogously to all other fungi [12]. As anaerobic fungi do not experience oxidative stress from ultraviolet light in their native conditions, it is possible that melanin produced by anaerobic fungi would instead insulate them from oxidative stress from reactive oxygen species formed during the ingestion and rumination of plant material by the host herbivore.

In this study, we characterize the metabolomic and transcriptomic response of the anaerobic fungus *Neocallimastix californiae* to alkaline lignin. While alkaline lignin is a non-naturally occurring byproduct of lignocellulose processing, it provides a useful experimental tool to separate fungal responses to carbohydrates from responses to aromatic compounds in a defined medium. We observe that *N. californiae* shifts its transcriptome in response to lignin-derived aromatics, differentially regulating genes associated with amino acid metabolism and producing melanin. These transcriptional changes correspond with shifts in the metabolic end products of the fungus as well as the removal of vanillin from the fungal growth media. These results suggest that anaerobic fungi can be metabolically stimulated by the aromatic fractions of lignocellulose, likely through an ability to scavenge building blocks for amino acids.

## Methods

### Cultivation of *Neocallimastix californiae* in defined (M2) medium to interrogate central metabolism

The anaerobic fungus *Neocallimastix californiae* [15, 16] was cultivated under a variety of conditions in defined M2 medium [39] with varying carbon sources of alkaline lignin (at two different concentrations) and/or cellulose, as Whatman paper cut into 5-mm-wide strips at a concentration of 10 g/L. All conditions received 0.22 µm filtered amendments of a vitamin solution and heme (protoporphyrin IX) at the concentrations previously described [40, 41]. All cultures were initially 40 mL in liquid volume and were grown in 80 mL serum bottles sealed with butyl rubber stoppers and crimped closed with aluminum caps.

Alkaline lignin was purchased from Fisher Scientific (TCI America, Portland, OR, USA, Part No. L0082) and added to cultivation medium before autoclaving in cases where 2.5 g/L of lignin extract was included (Additional File 2). In the low lignin addition condition, a concentrated stock solution of alkaline lignin was 0.22 µm filtered and added to cultivation medium after autoclaving to a final concentration of 0.125 g/L.

Fungal metabolic activity was monitored in these cultures using the pressure transducer technique as previously described [42], and samples for monitoring aqueous and gaseous metabolites were taken at each time point. For aqueous metabolites, 1 mL of culture was withdrawn and frozen for subsequent high-performance liquid chromatography (HPLC) and liquid chromatography–mass spectrometry (LC–MS) analysis. Further, 1 mL of headspace gas was withdrawn at each time point and analyzed by gas chromatography (GC). After sampling headspace gasses, pressure in the culture headspace was vented to 1.0 PSIG.

At the final timepoint of fungal growth, cultures were harvested to interrogate the mass of cellulose remaining in the culture. The remaining volume of culture was poured into a 50-mL Falcon tube and lyophilized for 2 d on a Labconco Freezone 4.5 L Benchtop lyophilization system (part no. 77500200, Labconco). The mass of cellulose depolymerized by the fungus during growth was calculated by subtracting the mass remaining in the culture vessel from the mass added to the culture vessel.

### Measurement of *N. californiae* metabolic activity with HPLC and total pressure accumulation

Total fermentation gas accumulation was monitored daily using the pressure transducer technique (PTT) to estimate fungal growth [19, 42]. After measuring daily pressure accumulation, headspaces were vented to 1.0 psig.

We further quantified fungal metabolites as a secondary metric of fungal activity using HPLC as previously described [40, 41]. Samples were run on a 1260 Infinity HPLC (Agilent Technologies, Santa Clara, CA, USA) equipped with an Aminex HPX-87H analytical column (part no. 1250140, Bio-Rad, Hercules, CA, USA). Separation conditions were 0.6 mL min^−1^ at 50 °C, and a 5 mM sulfuric acid (H_2_SO_4_) mobile phase was used. The refractive index detector was set to 35 °C, and the variable wavelength detector was set to 210 nm. Guard columns were a 0.22 µm physical filter, followed by a Coregel USP L-17 guard cartridge (Concise Separations, San Jose, CA, USA). Compounds monitored were acetate, formate, succinate, ethanol, lactate, cellobiose, and glucose. All HPLC standards of 0.1%, 0.05%, and 0.01% (w/v) were prepared in M2 Medium to account for medium background [41]. During analysis, blank medium chromatograms were subtracted from all standard and experimental chromatograms using OpenLab CDS analysis software (Agilent Technologies).

Samples and standards for HPLC were prepared by acidification to a concentration of 5 mM H_2_SO_4_, incubated for 5 min at room temperature, and spun at maximum speed in a tabletop centrifuge for 5 min to pellet fungal cells, proteins, and other debris. The acidified samples were removed from above the pellet and 0.22 µm filtered through a polyethersulfone (PES) membrane into HPLC vials with 300 µL polypropylene inserts.

### Analysis of hydrogen using GC

Hydrogen gas production was measured on a Fisher Scientific TRACE 1300 Gas Chromatograph (Thermo Fisher Scientific, Waltham, MA) using a TRACE TR-5 GC Column (part no. 260E113P, Thermo Fisher Scientific) at 30 °C, with an Instant Connect Pulsed Discharge Detector (PDD) (part no. 19070014, Thermo Fisher Scientific) at 150 °C, and ultra-high purity He as a carrier gas. All injections of samples and standards were 100 µL in volume. Supplier-mixed standards of 1%, 3%, 5%, 10%, and 20% hydrogen (balance helium) were run before and after injecting samples, and hydrogen peaks were integrated using Chromeleon Chromatography Data System (CDS) Software (Thermo Fisher Scientific).

### Analysis of monoaromatics in cultivation medium using LC–MS

Samples for LC–MS were prepared by filtering *N. californiae* supernatants through 0.22 µm PES membranes, then through 3000 Da molecular weight cutoff, PES, centrifugal filter units (12,000 × g, 30 min, 20 °C). The filtrate from the 3000 Da centrifugal filter was diluted with one part HPLC grade methanol (v/v). Analytical standards of aromatic compounds, samples, and uninoculated controls were analyzed using an Agilent Technologies HPLC-ESI-TOF–MS [43]. Calibration curves were used for the absolute quantification of each analyte of interest. The theoretical *m*/*z* of the deprotonated analytes was used to identify the aromatics of interest. Aromatics of interest were *p*-coumaric acid (163.040068), ferulic acid (193.050632), *p*-hydroxybenzoic acid (137.024418), vanillin (151.040068), vanillic acid (167.034982), syringic acid (197.045547), caffeic acid (179.034982), protocatechuic acid (153.019332), catechol (109.029503), and salicylic acid (137.024418).

### Cultivation conditions and harvesting protocol for RNA samples for RNA-Seq

Cultivation conditions for the RNA-Seq experiment followed those previously described. Cultures were grown in 40 mL of M2 medium and loaded with 10 g/L of Whatman paper strips (cellulose), and for the experimental condition, only 0.125 g/L of alkaline lignin (Fig. S5). Alkaline lignin was dissolved in MilliQ water and 0.22 µm filtered to avoid autoclaving the amendment. Six cultures and a single uninoculated control were started for each condition, and bottles were sampled for pressure daily using the PTT method. Three of the 6 cultures were earmarked for RNA harvest, and 3 were earmarked for continued monitoring of gas production, cellulose deconstruction, and liquid metabolites (Additional File 3).

To harvest cultures for RNA extraction, 1 volume of culture was added to 2 volumes of RNAlater in a sterile bottle. RNAlater was then removed from the sample by vacuum filtering biomass and remaining cellulose onto a glass fiber filter (part no. 09-804-24C, Fisher Scientific). Samples, including the glass fiber filter, were placed in a 5-mL Eppendorf tube and immediately flash-frozen in liquid nitrogen.

To extract RNA from frozen samples, including the glass fiber filters, were ground to a fine powder in liquid nitrogen using a mortar and pestle and immediately immersed in 1.5 mL of RLT buffer from the Qiagen RNAeasy kit (Qiagen, part no. 74004). Extraction then followed the protocol for infected tissue. Specifically, the slurry of cellulose and fungal biomass was pelleted at 4000x*g*, and 750 µL RLT buffer containing RNA and fungal cells was removed from the tops of the 5-mL Eppendorf tube and passed through a QIAshredder column. The subsequent RNA purification followed the standard RNAeasy protocol using an on-column DNase digestion. RNA extractions were then checked for yields and quality using a Qubit fluorometer (ThermoFisher Scientific, Waltham, MA, USA) and a Tapestation microfluidic electrophoresis device (Agilent Technologies, Santa Clara, CA, USA).

### Sequencing library construction

Approximately 2 µg of high-quality mRNA was separated from rRNA using the NEBNext Poly(A) mRNA Magnetic Isolation Module, and this mRNA was subsequently used to construct sequencing libraries (New England Biolabs, Ipswich, MA, USA, part no. E7490S). Stranded libraries were then synthesized using a NEBNext Ultra II Directional Library Prep Kit for Illumina (New England Biolabs, part no. E7760S). Libraries were sequenced on an Illumina NextSeq 500, using a NextSeq 500/550 High Output Kit v2.5 (300 Cycles) kit (Illumina part No. 20024908), in the Biological Nanostructures Laboratory at the University of California Santa Barbara.

### Differential expression analysis of transcriptomic data

Reads obtained from sequencing were filtered for quality using BBDuk, resulting in 140 million reads that were suitable for alignment to the reference genome [44]. Individual libraries ranged between 20 and 27 million high-quality reads. Quality-controlled reads were subsequently aligned to the appropriate reference genome (Neosp1_AssemblyScaffolds.fasta.gz) using HISAT2 [45, 46]. Alignment by HISAT2 produced an average alignment rate of 93.8% for the six libraries. The alignments produced by HISAT2, along with an appropriate genome feature file (Neosp1_GeneCatalog_genes_20170918.gff.gz), were next used to generate raw counts of transcripts using featureCounts [47]. Both the genomic assemble and genomic feature file used are available on The Joint Genome Institute’s Mycocosm database (https://mycocosm.jgi.doe.gov/mycocosm/home) [25]. The raw counts were then used as the input matrix for differential expression analysis by DESeq2 [48].

### Melanin extraction and quantification

Cultures were grown under the previously described conditions where one set of triplicate cultures received pure cellulose to serve as a control, while a second set of three cultures received both cellulose and 0.125 g/L of alkaline lignin. At the cessation of growth, cultures were pelleted using centrifugation and dried in a Freezone 4.5-L Benchtop lyophilization system.

To extract and purify melanin from the freeze-dried pellets of fungi, we designed a protocol based on suggestions supplied with the Amplite^®^ Fluorimetric Melanin Assay Kit from AAT Bioquest (Part No. 11310). Pellets were rehydrated in 30 mL of MilliQ water for 5 min, and then these tubes were brought to a boil for 5 min in a beaker of boiling water. Samples were then placed in a sonicator bath for 5 min to improve cell disruption. After sonication, 18 mL of 1 M NaOH was added to each tube, and the tubes were sealed. The 50-mL Falcon tubes were then loosely capped to allow venting and autoclaved (121 C, 20 PSI) for 20 min on a liquid cycle. After autoclaving, these cell lysates were filtered through a 25 mm glass fiber filter (MilliporeSigma, Part No. AP4002500) to obtain a clarified lysate containing soluble melanin. Melanin was then precipitated by titrating each tube with 6 M HCl until the pH was between 1 and 2. These samples were then re-filtered through No. 1 Whatman paper filters (Cytiva Whatman, Part No. 1001–325) backed by a 0.45 µm mixed cellulose ester backing filter (MilliporeSigma, Part No. GSTF02500) and washed three times with sterilized, 0.22 µm filtered MilliQ water. The washed Whatman paper filters were then dried at ambient conditions overnight before precipitated melanin was extracted from filter surfaces by immersing them in 1 mL of DMSO for 10 min. The melanin content in each sample was then quantified using the Amplite^®^ Fluorimetric Melanin Assay Kit from AAT Bioquest.

## Results

### Fungal cultivation and cellulose deconstruction

The anaerobic fungus *Neocallimastix californiae* was grown in defined M2 medium with cellulose as a substrate, and alkaline lignin (AL) was added at several concentrations to determine the nature of interactions between the anaerobic fungus and aromatic chemicals. It is important to note that although alkaline lignin is derived from lignin, it is heavily derivatized and fragmented during its extraction and therefore no longer resembles lignin and does not closely resemble the immediate breakout products of lignin. Despite the limited overlap between monoaromatics and oligomers obtained from naturally occurring lignin degradation processes, we note that our 0.125 g/L supplementation is physiologically relevant in natural settings since the total concentration of all aromatics in solution is one that could be realistically obtained from the dissolution of lignocellulose if loaded at 10% w/v. Given the differences between alkaline lignin and natural lignin degradation products, we find the results we obtained to be most useful in terms of deconvoluting the relationship between aromatic chemicals and lignin and less useful for understanding how anaerobic fungi interact with lignin in natural systems.

**Fig. 1:**
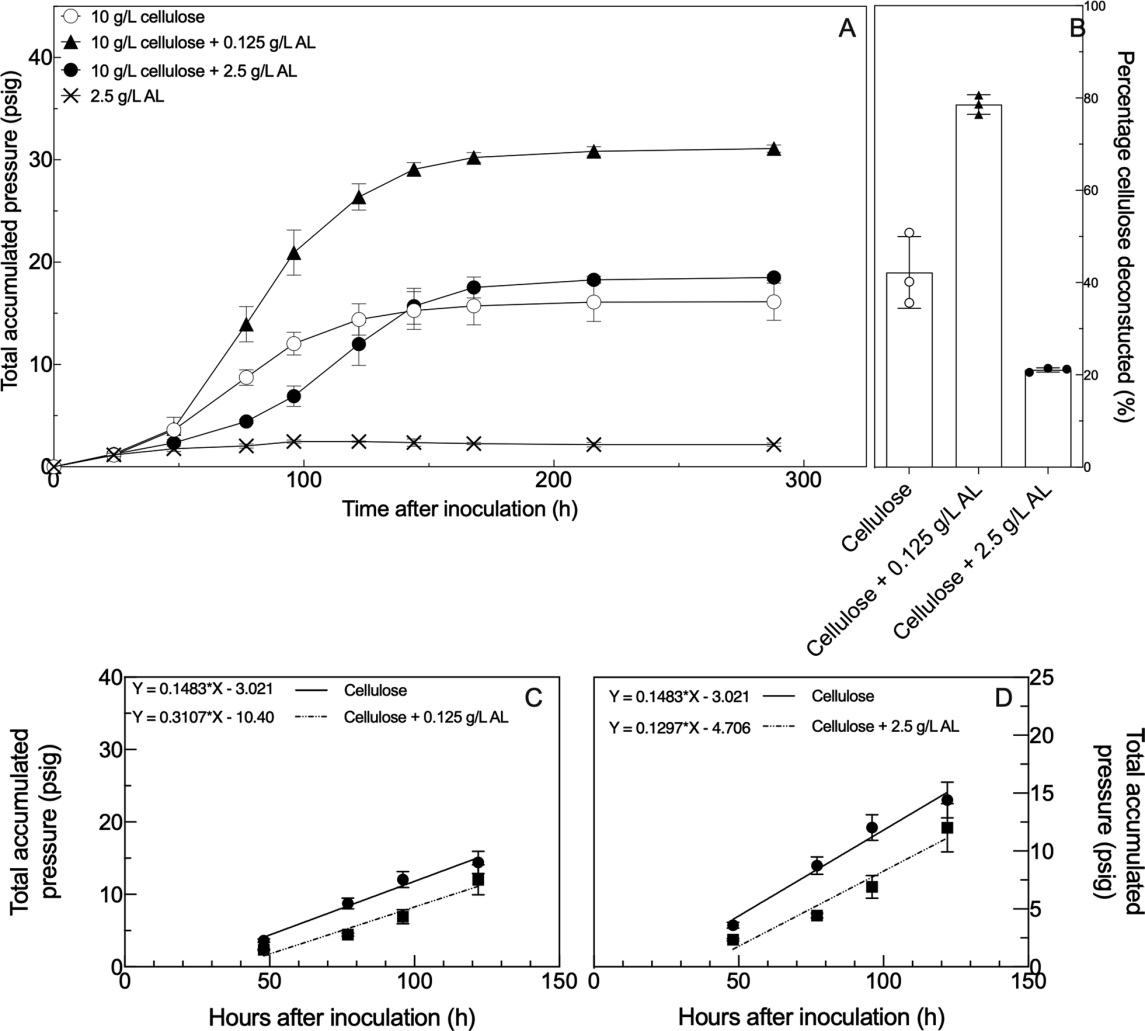
Pressure production and extent of cellulose deconstruction for *N. californiae* in defined M2 medium vary with the addition of alkaline lignin (AL) extract. Panel **A** shows pressure production over time as measured using the pressure transducer technique (PTT). Panel **B** depicts the amount of cellulose deconstructed by each culture type. In Panel **B**, both mean and individual values are shown. Panels **C** and **D** represent ANCOVA comparisons between the steady-state portions of culture pressure production, which were estimated to be between 48 and 122 h. Panel **C** represents the comparisons between the cellulose-only culture and the culture loaded with both cellulose and 0.125 g/L alkaline lignin. Panel **D** represents the comparisons between the cellulose culture and the culture loaded with both cellulose and 2.5 g/L alkaline lignin. In all panels, error bars represent the standard deviation of biological replicates (*n* = 3). In all panels AL abbreviates “alkaline lignin”

At 0.125 g/L, AL enhanced fungal metabolic activity (Fig. 1) as judged by fermentation gas production (a proxy of fungal growth [42]). The 0.125 g/L addition increased the rate of gas production (ANCOVA, *p* < 0.0001, Fig. 1) and the final pressure yield (Student’s t-test, *p* < 0.0001, Fig. 1). When 2.5 g/L of AL (a higher concentration that the fungus might experience from normal lignocellulose dissolution) was added to cultures, the rate of gas production did not change (ANCOVA, *p* = 0.3454), but these cultures had longer lag phases than control cultures containing only cellulose (ANCOVA, *p* < 0.0001). The cellulose + 2.5 g/L alkaline lignin amendment condition produced a similar total pressure (18.5 psig) as the culture containing only cellulose (16.1 psig). Additionally, the fungus did not use 2.5 g/L AL as a sole carbon source in M2 medium, as demonstrated by the lack of growth when AL was supplied as the sole carbon source (Fig. 1).

The addition of AL affected the ability of the fungus to deconstruct cellulose. This effect depended on the amount of alkaline lignin added (Fig. 1). Control cultures with no AL amendment deconstructed 40% of the supplied cellulose. In contrast, cultures amended with 0.125 g/L alkaline lignin could deconstruct 80% of the cellulose provided. Alkaline lignin additions at 2.5 g/L were, on the other hand, inhibitory, reducing the amount of cellulose deconstructed to 20%. All these differences between the amounts of cellulose deconstructed were statistically significant as determined using Student’s t-test (*p* < 0.001). These differences in deconstruction are also reflected in the concentrations of sugars released from cellulose, where more deconstruction results in a greater concentration of reducing sugars (Additional File 1).

**Fig. 2:**
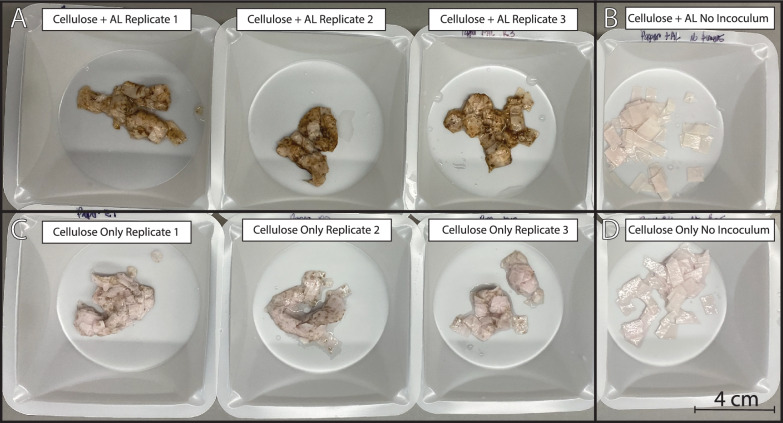
Pellets of cellulose with attached fungal biomass harvested from cultures grown in M2 medium, on cellulose, with and without 0.125 g/L of alkaline lignin (AL), demonstrate browning in the 0.125 g/L alkaline lignin condition but not in the control condition. Panel **A** contains images of cellulose pellets where cellulose + 0.125 g/L of alkaline lignin was added to the medium, while the companion uninoculated control is shown in Panel **B**. Panel **C** depicts cellulose-fungus pellets where only cellulose was supplied in the growth medium; the companion uninoculated control is shown in Panel **D**. The fungal biomass is visible as an off-white to yellow color in the cellulose-only cultures, whereas fungal biomass is more distinctly visible as the brown substance coating cellulose in the cellulose + AL cultures. Cellulose (as Whatman paper) remains intact in uninoculated controls, maintaining its initial rigidity. In inoculated cultures, the cellulose has been partially depolymerized, causing the strips of Whatman paper to lose their shape and rigidity

The varied metabolic responses to the differing carbon sources were accompanied by a striking, macroscopic visual observation. In the presence of only cellulose, fungal biomass possessed an off-white to yellow color. However, when 0.125 g/L AL was supplied in addition to cellulose, the fungal biomass appeared dark brown (Fig. 2). The no inoculation controls that accompanied live cultures did not produce the same browning when AL was added, indicating that adsorption of AL to cellulose was not responsible for the observed color change. Given the lack of color changes in abiotic controls, we interpret this observation as a biological phenomenon where the fungus has mediated a biochemical change to the composition of its cell wall, causing observable browning in the presence of 0.125 g/L AL.

**Fig. 3:**
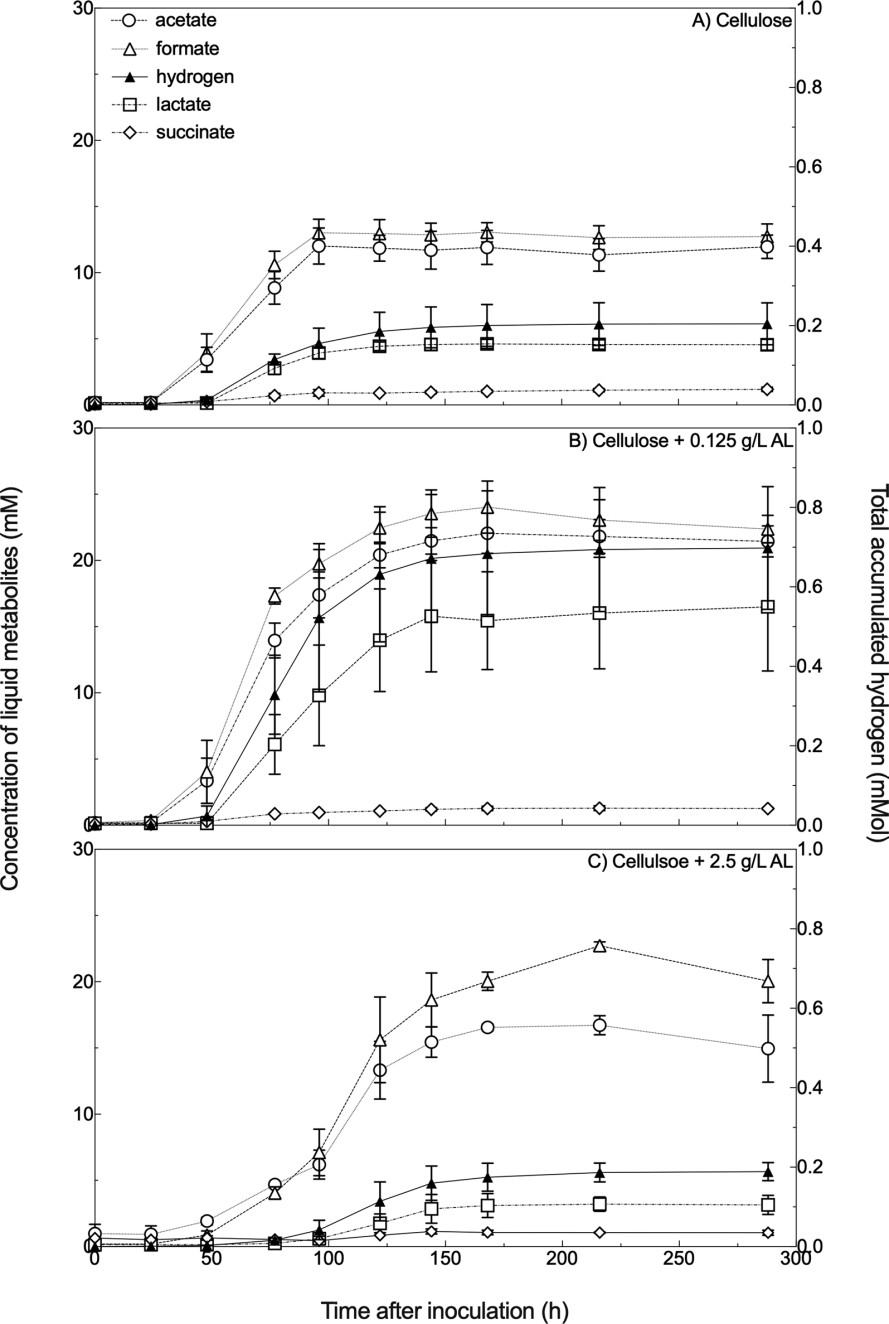
Short-chain fatty acid and hydrogen gas production by *N. californiae* in defined M2 medium change with the addition of alkaline lignin (AL). Panel **A** shows metabolites in the cellulose-only cultures, Panel **B** shows metabolites in cultures with cellulose and 0.125 g/L alkaline lignin. All liquid metabolites are graphed in millimolar concentrations, shown on the left Y axis. Hydrogen is represented as the total amount of hydrogen produced in millimoles, shown on the right Y axis. In all panels, error bars represent the standard deviations of biological replicates (*n* = 3)

Examining the metabolic products of fungal activity revealed that the increased growth rate and yield in the presence of 0.125 g/L AL resulted from increased carbon flow through central metabolism (Fig. 3). The ratio of fungal metabolites, especially the ratio of formate to acetate, can inform which of their two encoded central metabolic pathways is being actively employed to process carbon [20]. At the end of growth, the ratio of formate to acetate (moles formate/moles acetate) in the 0.125 g/L AL-supplemented cultures and the cellulose control was approximately one but increased to 1.34 for the 2.5 g/L AL-supplemented cultures. Amounts of lactate normalized to the amount of acetate (moles lactate/moles acetate) were twice as high in the 0.125 g/L AL-supplemented condition (0.76) as in the controls (0.38) and in the 2.5 g/L AL condition this ratio was lower than in the controls (0.21). Hydrogen is produced in anaerobic fungal cultures in much lower molar amounts than the short-chain fatty acids. Still, the ratio of this metabolic product to acetate (moles hydrogen/moles acetate) also changed in the 0.125 g/L AL-supplemented condition. The hydrogen to acetate ratio in the control cultures was 0.017, and it nearly doubled to 0.032 in the 0.125 g/L condition but fell to 0.013 in the 2.5 g/L condition. The ratio of succinate to acetate (moles succinate/moles acetate) was 0.1 in the control cultures but 0.6 in the cultures supplemented with 0.125 g/L AL and 0.7 in the 2.5 g/L AL-supplemented cultures.

### Concentrations of detectable aromatics

**Fig. 4:**
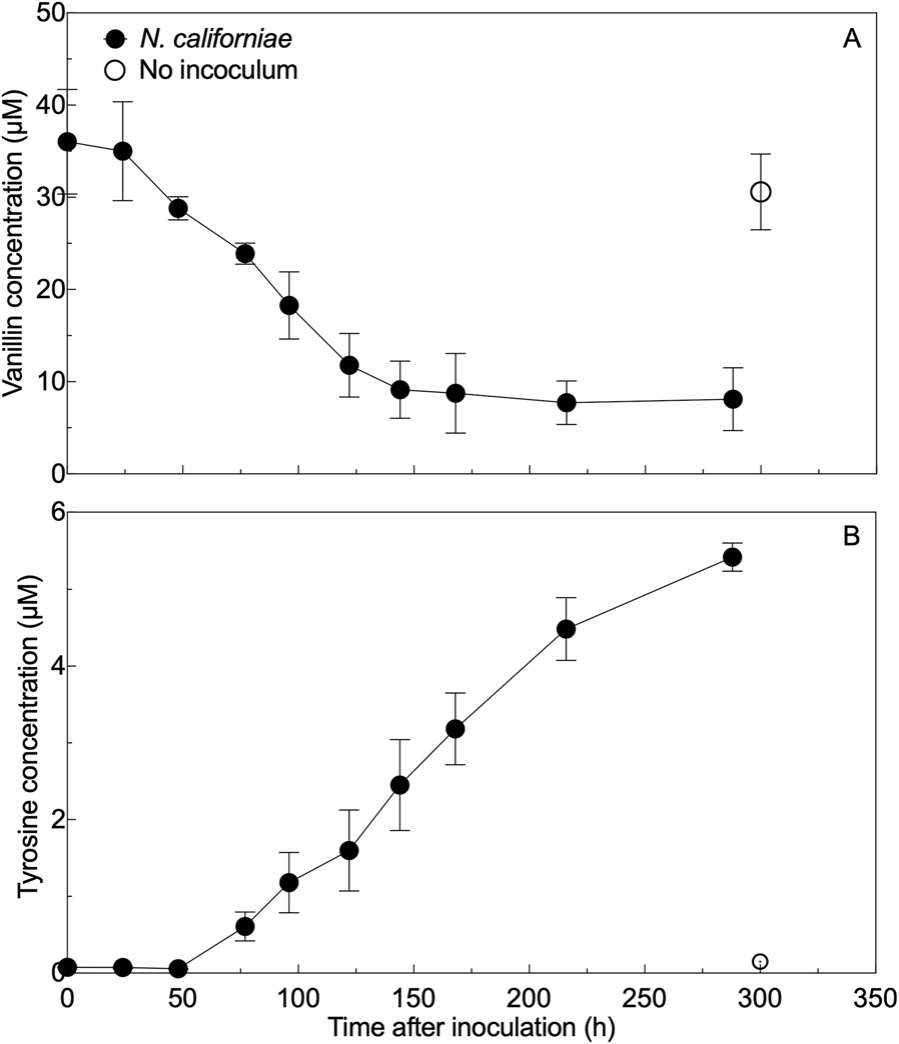
*N. californiae* draws down vanillin and, shortly after beginning to remove vanillin from the media, begins producing tyrosine. Measurement by LC–MS reveals two aromatic compounds with distinct trends in their concentrations over the course of *N. californiae* growth in the presence of cellulose and 2.5 g/L alkaline lignin. Panel A shows the concentration of vanillin over time, while Panel B shows the concentration of tyrosine over time. The error bars in both panels represent the standard deviations of biological replicates (*n* = 3)

Data collected via LC–MS indicate that shifts in *N. californiae* metabolism coincided with changes in quantifiable aromatics in the cultures with 2.5 g/L alkaline lignin added (Fig. 4). The concentration of individual aromatic compounds was below the limit of quantification in the 0.125 g/L AL-supplemented cultures. In the 2.5 g/L AL-supplemented cultures also containing cellulose, fungi appeared to consume vanillin and produce tyrosine. It is unclear from these chemical data whether this is an extracellular process or if the fungus is transporting vanillin into cells for conversion. The accumulated tyrosine is approximately 1/6 of the molar amount of vanillin consumed. Therefore, any ongoing aromatic processing is more complex than a simple 1:1 molar conversion of vanillin to tyrosine. The evolution of tyrosine implied that a transcriptomic response to alkaline lignin might be observed in genes related to amino acid metabolism.

### Transcriptomic remodeling in the presence of alkaline lignin

Transcriptomic data obtained from *N. californiae* grown on cellulose compared to those from *N. californiae* grown on cellulose with an addition of 0.125 g/L alkaline lignin demonstrated gene regulation signatures of interest (Table 1). Five genes of interest related to amino acid metabolism were differentially regulated. Three copies of ketol-acid reductoisomerase (E.C. 1.1.1.86), an enzyme involved in valine, leucine, and isoleucine biosynthesis, were downregulated. In contrast, two upregulated genes were associated with amino acid metabolism: a single glutamate dehydrogenase (E.C. 1.4.1.4) and an anthranilate synthase (E.C. 4.1.3.27). Five additional genes related to central metabolism were also upregulated; three copies of a phosphoenolpyruvate carboxykinase (E.C. 4.1.1.32) were expressed more in the condition with alkaline lignin, as were single copies of an acetyl-CoA hydrolase (E.C. 3.1.2.1) and malate dehydrogenase (E.C. 1.1.1.40). A single gene coding for an aryl-alcohol dehydrogenase (E.C. 1.1.1.91) implicated in the transformation of aromatic alcohols was downregulated. Notably, some other genes associated with central metabolism in anaerobic fungi, such as lactate dehydrogenases, hydrogenases, pyruvate formate lyases, and pyruvate formate oxidoreductase, were not differentially regulated in the condition with added alkaline lignin. Of the 11 genes highlighted here, 0 of them had signal peptides, and 0 had detectable transmembrane domains. The gene identified as a malic enzyme had a small hydrophobic region identified as a hydrogenosome localization peptide.

**Table 1:** Genes of interest identified using differential expression analysis are implicated in central metabolism, amino acid metabolism, and processing of aromatic carbon. The table below shows relevant information for selected differentially expressed transcripts of interest. Log2fold changes, transcript counts, and adjusted *p*-values were calculated using DESeq2. Transcript IDs and annotations are supplied by the Joint Genome Institute’s Mycocosm database (https://mycocosm.jgi.doe.gov/mycocosm/home)

Transcript ID	Enzyme name	Cellulose only transcripts	Cellulose + 0.125 g/L AL transcripts	log2Fold change	Lfc SE	padj	E.C	Relevant processes
430828	Phosphoenolpyruvate carboxykinase (GTP)	0.67 ± 0.58	37.33 ± 29.57	5.62	1.22	0.0004	4.1.1.32	Gluconeogenesis
503009	Phosphoenolpyruvate carboxykinase (GTP)	20.33 ± 7.51	219.33 ± 108.79	3.26	0.44	0.0000	4.1.1.32	Gluconeogenesis
701958	Phosphoenolpyruvate carboxykinase (GTP)	17.33 ± 8.39	75.67 ± 32.32	1.95	0.48	0.0033	4.1.1.32	Gluconeogenesis
391562	Malate dehydrogenase (oxaloacetate-decarboxylating) (NADP( +))	40,802.00 ± 6508.40	75,065.33 ± 6520.47	0.73	0.20	0.0146	1.1.1.40	Malate metabolic process
456019	Acetyl-CoA hydrolase	1417.00 ± 294.37	2957.67 ± 585.79	0.92	0.24	0.0061	3.1.2.1	Acetyl-CoA metabolic process
524274	Anthranilate synthase	1300.00 ± 299.77	2543.67 ± 214.87	0.82	0.22	0.0086	4.1.3.27	Tryptophan biosynthetic process
706036	Glutamate dehydrogenase (NADP( +))	2808.33 ± 360.97	5759.33 ± 555.02	0.89	0.21	0.0015	1.4.1.4	Amino acid metabolic process
453370	Aryl-alcohol dehydrogenase (NADP( +))	69.33 ± 22.68	28.00 ± 11.27	−1.48	0.40	0.0135	1.1.1.91	Aromatic alcohol transformation
191723	Ketol-acid reductoisomerase (NADP( +))	229.00 ± 66.00	125.67 ± 23.71	−1.03	0.30	0.0248	1.1.1.86	Branched-chain amino acid biosynthetic process
386165	Ketol-acid reductoisomerase (NADP( +))	186.67 ± 47.51	91.00 ± 25.12	−1.20	0.32	0.0101	1.1.1.86	Branched-chain amino acid biosynthetic process
523478	Ketol-acid reductoisomerase (NADP( +))	124.00 ± 36.72	64.00 ± 8.19	−1.11	0.33	0.0331	1.1.1.86	Branched-chain amino acid biosynthetic process

### Measurement of melanin concentrations in fungal biomass

**Fig. 5:**
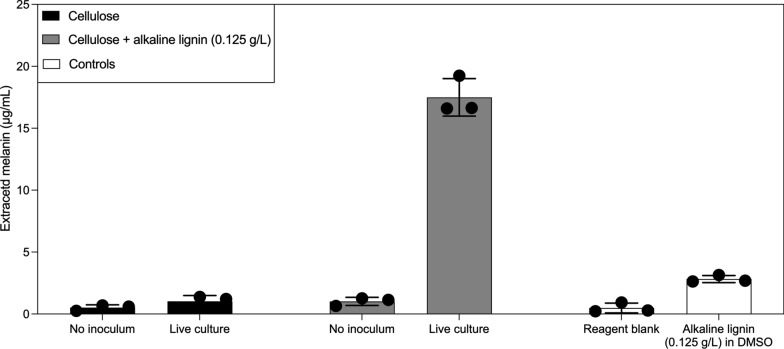
Anaerobic fungi produce melanin when exposed to 0.125 g/L alkaline lignin (AL). Extracting melanin from cultures grown with and without 0.125 g/L alkaline lignin demonstrates that anaerobic fungi produce melanin when exposed to aromatics but not under conditions where alkaline lignin is absent. Reagent blank samples were subjected to the melanin extraction protocol in parallel with biological samples and uninoculated controls to account for any background fluorescence. Samples with alkaline lignin (0.125 g/L) in DMSO were created to demonstrate that alkaline lignin itself does not interfere with the melanin concentration assay. Error bars represent the standard deviations of biological replicates (*n* = 3)

In cultures supplemented with AL additions, we repeatedly observed browning of fungal biomass, which suggested to us that either AL was being adsorbed onto fungal cells or the fungi were producing melanin, the most common explanation for the browning of fungal cells. Since tyrosine, which we observed accumulating in the fungal growth media, is a downstream precursor of melanin, we tested fungal biomass, grown under disparate conditions, for melanin content. Melanin was present in detectable concentrations in cultures exposed to cellulose and 0.125 g/L of alkaline lignin but not in cultures that were only grown on cellulose (Fig. 5). This measurement complements the observed gene regulation events described above and the observation that fungal biomass turns brown in the presence of 0.125 g/L alkaline lignin (Fig. 2). Samples extracted from uninoculated controls, but with AL added, demonstrated no extractable melanin above the assay background, and AL at 0.125 g/L did not produce a false positive in the melanin assay.

## Discussion

Anaerobic fungi evolved to depolymerize the diverse polymers contained in lignocellulose. Aromatic moieties are found in abundance in lignin, which anaerobic fungi were recently discovered to deconstruct [17], but also in hemicellulose sidechains [7]. It has often been assumed that the role of aromatic moieties in interactions between anaerobic fungi and lignocellulose is antagonistic. Aromatics are commonly expected to protect polysaccharides through steric hindrance and inhibition of CAZymes, but this contrasts with the established knowledge that some anaerobic bacteria use available aromatic compounds [23, 24]. Here, we report evidence of anaerobic fungi improving utilization of cellulose in response to available aromatic compounds derived from lignin in a defined medium, expanding on understanding Neocallimastigomycetes’ interactions with the aromatic fractions of lignocellulose.

Alkaline lignin supplied to cultures, alongside cellulose, at a physiologically relevant concentration (cellulose + 0.125 g/L AL) enhanced fungal growth yield and growth rate, as measured with the pressure transducer technique, leading to twice as much pressure production at twice the rate of the control cultures. At 20-fold increased concentration, 2.5 g/L AL (cellulose + 2.5 g/L AL) reduced growth rate, but cultures reached the same final pressure yields as those cultures that had no alkaline lignin added to them. Data collected via HPLC and GC agree with the assessments of fungal growth rate and yield as determined by PTT. Concentrations and rates of production for acetate, lactate, formate, and hydrogen are all higher in the 0.125 g/L AL-supplemented condition. These data suggest that the fungi in the cellulose + 0.125 g/L AL amended cultures were able to shunt more carbon through central metabolism, but that this same mixture, supplied at a 20-fold increased concentration, was unable to support growth by itself. Additionally, the formate/acetate ratios in these three conditions suggest that the fungus shunted carbon through pyruvate formate lyase in the control and cellulose + 0.125 g/L AL-supplemented conditions, giving a ratio of 1:1. Conversely, more carbon may have been shunted through the alternate pyruvate formate oxidase pathway in the cellulose + 2.5 g/L AL condition, giving a formate/acetate ratio of 1.34 [20]. We interpret these results as evidence that alkaline lignin additions enhance fungal metabolism, but anaerobic fungi are unable to use 2.5 g/L AL as their sole carbon source.

Some anaerobic bacteria that evolved in the presence of lignocellulose catabolize aromatic monomers resulting from the deconstruction of lignin [23, 24]. Anaerobic bacteria of various lineages encode two distinct pathways of aromatic catabolism, one of which costs two ATP per aromatic monomer processed, and a second with no reducing equivalent cost [23, 24]. Both pathways produce three acetyl-CoA, which can subsequently be processed by fermentative metabolism to yield three ATP. These two pathways start with benzoyl-CoA, but companion pathways termed “peripheral pathways” convert diverse aromatic monomers into benzoyl-CoA [23]. Given our novel observation in cultures of anaerobic fungi, we rechecked the *N. californiae* genome for any potential homologs of these pathways, but still found none [25].

Crude deconstruction measurements using the mass of cellulose before and after growth support the finding that cultures with cellulose and 0.125 g/L of alkaline lignin were more active. The amount of cellulose deconstructed in the cellulose + 0.125 g/L alkaline lignin supplemented condition (80% by weight) was double that of the control culture (40% by weight), which in turn was double the amount of cellulose deconstructed in the cellulose + 2.5 g/L AL condition (20% by weight). The control culture with no AL added deconstructed less cellulose than cultures of *N. californiae* grown in complex media, which deconstructed 90% of cellulose during growth, as reported previously [49]. The disparity between complex medium deconstruction (90%), defined medium deconstruction (40%), and defined medium amended with 0.125 g/L alkaline lignin suggests that anaerobic fungal cultures benefit to an extent from the presence of non-carbohydrate carbon in the growth media. The cellulose + 2.5 g/L alkaline lignin supplemented condition produced the same amount of metabolic activity as the cellulose control, but this metabolic activity only resulted in half the cellulose deconstruction of the cellulose-only condition. Similar growth yields in this condition, coupled with less carbohydrate consumption, hint, but cannot confirm, that there may be anabolic assimilation of aromatic carbon in this condition.

Observations obtained using LC–MS also suggest that anaerobic fungi interact with the aromatic compounds found in alkaline lignin (Fig. 4). The observed drawdown of vanillin throughout growth is coupled with the appearance of tyrosine in the fungal growth medium in the 2.5 g/L condition. Despite the co-occurrence of these events, the maximum molar amounts of these two aromatic compounds differed by a large amount, indicating that while increased production of tyrosine might be a consequence of available aromatic carbon, it is likely not the only consequence. The appearance of tyrosine suggested that amino acid metabolism might be one potential area where a biological response, like transcriptome remodeling, could be observed. We were unable to collect data on the changes in concentration of these compounds in the cellulose + 0.125 g/L AL-supplemented condition because the concentrations of these individual aromatics were always below the method limit of quantification.

Transcriptomic data supported the hypothesis that anaerobic fungi shifted amino acid metabolism in response to the availability of 0.125 g/L alkaline lignin (Table 1). Specifically, we suspect that the presence of usable aromatic carbon in the growth medium allowed fungi to scavenge carbon for the synthesis of amino acids. Therefore, we intentionally sought out any genes that were putatively implicated in cellular processes related to aromatics or amino acids. We found 11 genes with differentially regulated transcripts that were implicated in these processes of interest. The increased abundance of transcripts coding for an anthranilate synthase, which was upregulated by over 1200 transcripts in the cellulose + 0.125 g/L AL condition (although the log2fold change was only 0.82), suggests that there was an increased cellular investment in the late stages of aromatic amino acid synthesis, while there was a decreased investment in early-stage synthesis as seen by the downregulation of three ketol-acid reductoisomerases. This switch in amino acid metabolism, the increased titer of primary fungal metabolites, increased pressure production, and increased cellulose deconstruction suggest the 0.125 g/L AL addition relieved growth inhibition by supplying amino acid building blocks. The suggested relief of growth inhibition was also accompanied by the upregulation of three PEP carboxykinases and a malic enzyme, further supporting the hypothesis that cells were able to shunt more carbohydrates through central metabolism when supplied with 0.125 g/L of alkaline lignin.

Transcriptomic signals, release of tyrosine into the fungal growth media, and observed browning of fungal biomass in the alkaline lignin amendment conditions (Fig. 2) all suggested that increased melanin synthesis, a well-documented fate of aromatic amino acids in fungal cells, might be observed. We successfully extracted and quantified melanin from fungal biomass in the 0.125 g/L alkaline lignin amendment condition but did not obtain any quantifiable melanin from cells in the carbohydrate-only condition (Fig. 5). We suggest this observation offers strong, additional evidence that anaerobic fungi are remodeling amino acid metabolism in the presence of 0.125 g/L alkaline lignin and that this modulation results in melanization of anaerobic fungal cells. While browning of these fungal cells has been previously observed, a detailed explanation for this phenomenon is still lacking [12, 20]. Melanin production is a consistent feature of organisms from the fungal kingdom, making this explanation logical [36, 37]. The suspected role of melanin in fungal cells is protection from environmental redox stress, which is often associated with stress from ultraviolet light in the fruiting bodies of high fungi [36, 37]. In this context, we suspect that anaerobic fungi evolved melanin synthesis to protect themselves from oxidative stress during oxygen intrusion into the rumen. Moreover, melanization may play a key role in developing an aerotolerant cyst stage of the fungi, which has been speculated to exist but not experimentally verified [19, 50–52].

## Conclusions

Anaerobic fungi not only deconstruct lignin, but also metabolically respond to soluble aromatics, although this shift in metabolism does not independently support growth in the absence of carbohydrates. It seems likely, but it is unconfirmed, that anaerobic fungi are taking up aromatic moieties of lignin and introducing them into anabolism as building blocks for aromatic amino acids. Scavenging of aromatic moieties subsequently might enhance fungal growth by allowing carbohydrates to be reserved for other cellular functions like energy conservation and synthesis of non-aromatic biomolecules. These results show that anaerobic fungi are prone to melaninization when aromatic resources are readily available and confirm that the deep-branching Neocallimastigomycetes Class produces melanin, like other fungal lineages. These results constitute the first evidence that anaerobic fungi might use aromatic chemicals derived from lignocellulose for amino acid synthesis and suggest that Neocallimastigomycetes could be valuable biotechnological tools for sensing and transforming aromatics as well as carbohydrates.

## Supplementary Information

Supplementary material 1.

## Data Availability

All data and materials contained in this manuscript are available from the corresponding author upon request. Sequences from transcriptomic experiments have been deposited in NCBI under BioProject: PRJNA1065920 with Accession #s: SRX23265111–SRX23265116.

## Abbreviations

CAZymes: Carbohydrate-active enzymes
HPLC: High performance liquid chromatography
GC: Gas chromatography
LC–MS: Liquid chromatography–mass spectrometry
AL: Alkaline lignin
ATP: Adenosine triphosphate
PTT: Pressure transducer technique
PES: Polyethersulfone
RNA: Ribonucleic acid
RNA-Seq: Ribonucleic acid sequencing
DMSO: Dimethyl sulfoxide
