# Supplementary material for: The anaerobic fungus Neocallimastix californiae shifts metabolism and produces melanin in response to lignin-derived aromatic compounds
Source: Biotechnol Biofuels Bioprod. 2025 Aug 29;18:96. doi: 10.1186/s13068-025-02696-5 (PMC12398058; doi:10.1186/s13068-025-02696-5)
Supplement: Supplementary file 1 — Supplementary material 1. [file 13068_2025_2696_MOESM1_ESM.docx]

**Supplementary Information: The anaerobic fungus *Neocallimastix californiae* shifts metabolism and produces melanin in response to lignin-derived aromatic compounds**

Authors: Thomas S. Lankiewicz^1,2,3^, Bashar Amer^3^, Edward E.K. Baidoo^3^, Patrick A. Leggieiri^1^, Michelle A. O’Malley*^1,3,4^

Affiliations:

^1^Department of Chemical Engineering, University of California Santa Barbara; Santa Barbara, CA, USA.

^2^Department of Ecology, Evolution, and Marine Biology, University of California Santa Barbara; Santa Barbara, CA, USA.

^3^Joint BioEnergy Institute, Lawrence Berkeley National Laboratory; Berkeley, CA, USA.

^4^Department of Bioengineering, University of California Santa Barbara; Santa Barbara, CA, USA.

* corresponding author: momalley@ucsb.edu

**Supplemental material:**


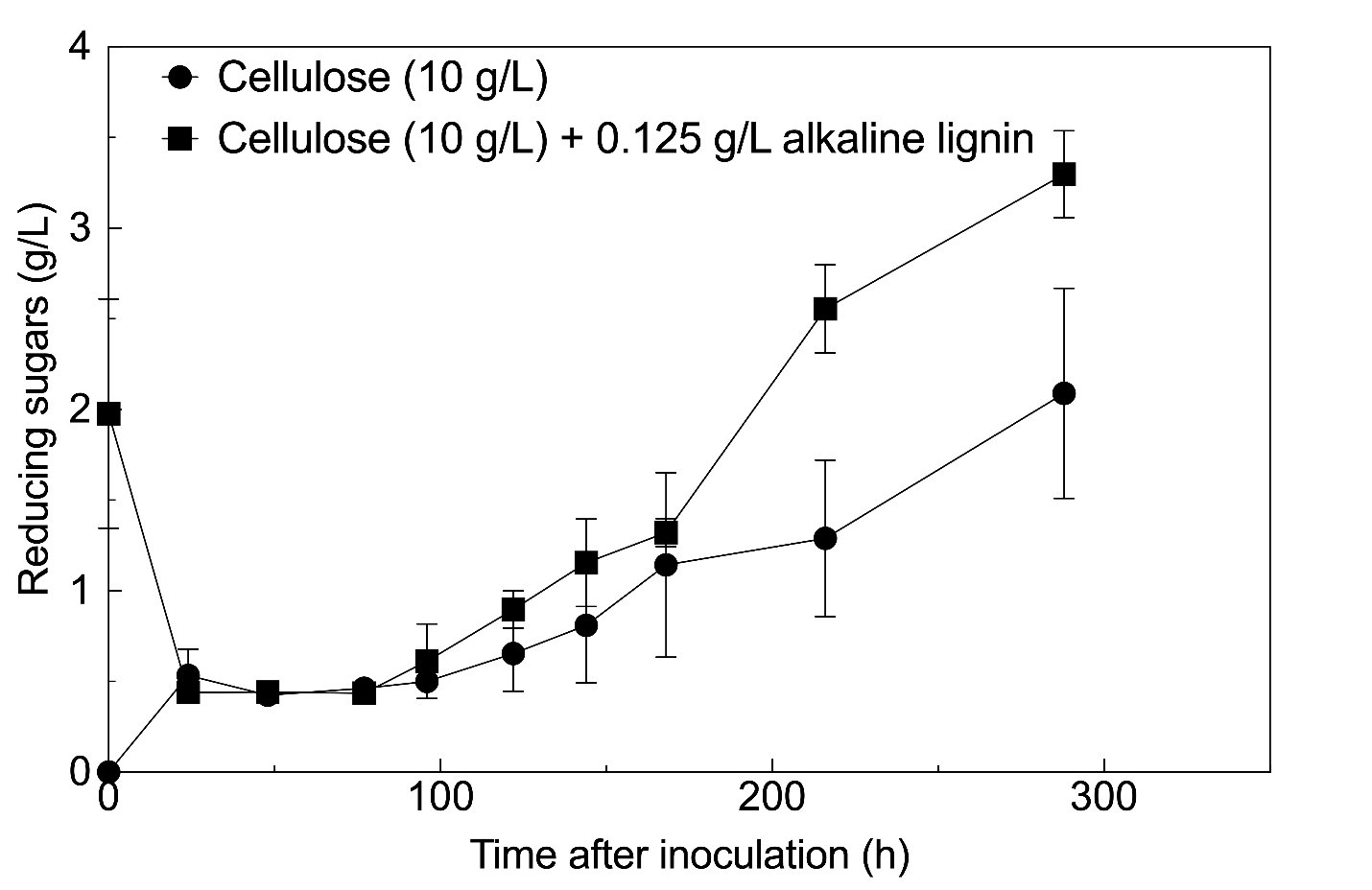


**Figure S1. Differences in excess sugars, freed from cellulose, reflect differences in deconstruction.** In panel A, reducing sugars for each timepoint are depicted for the cellulose-only treatment and the 0.125 g/L alkaline lignin treatment. In panel B, deconstructed extents determined by dry weight are depicted, and the same data is in Panel B in Fig. 1. In agreement with deconstruction data, the amount of reducing sugars is greatest in the 0.125 g/L alkaline lignin-added treatment and less in the control treatment with only cellulose added. Sugars were not measurable using the DNS assay in the 2.5 g/L treatment due to interference at 570 nm from low molecular weight aromatics. In all panels, error bars represent the standard deviation of biological replicates (n=3).


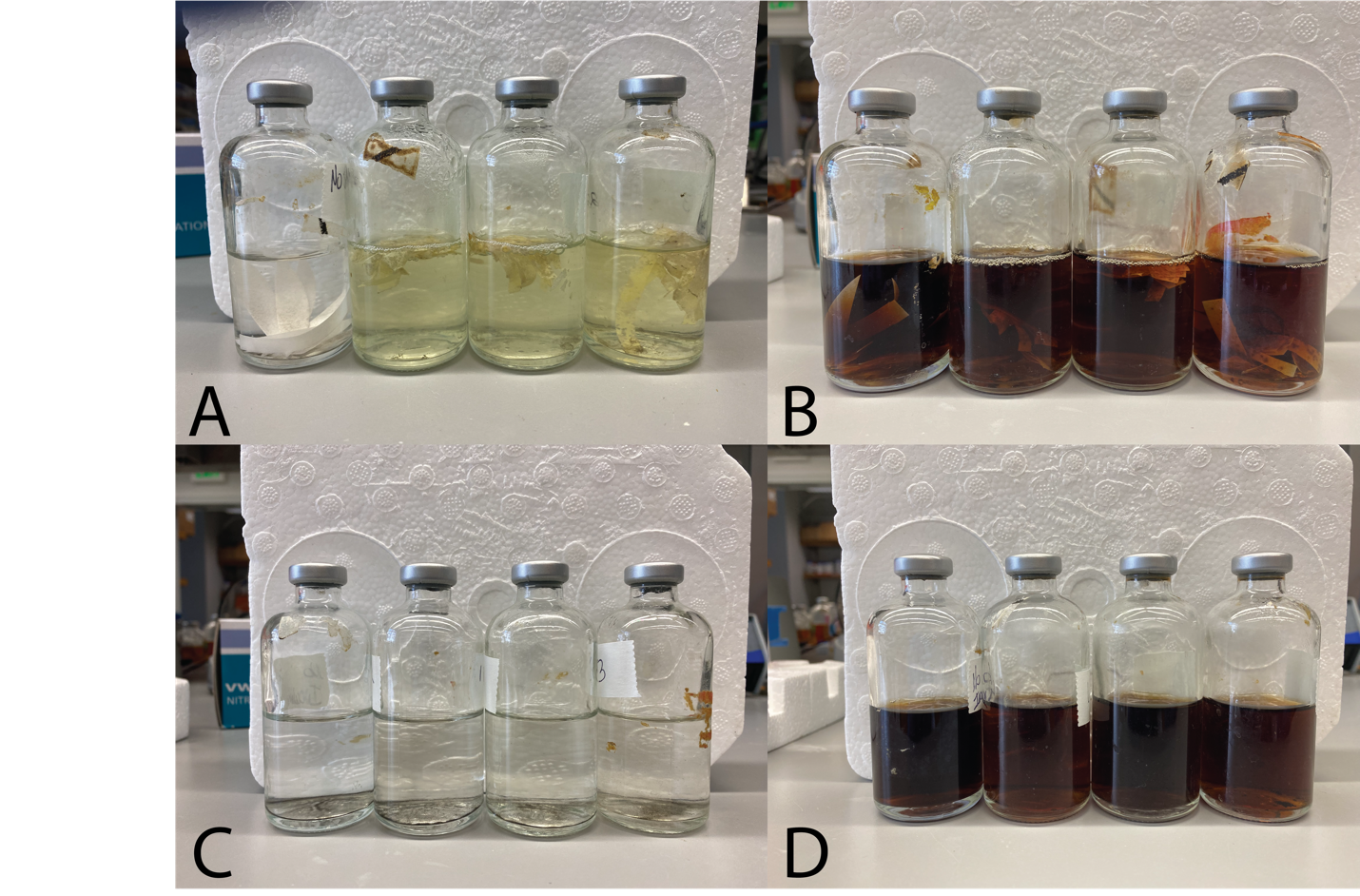


**Figure S2. Images of fungal cultures in M2 medium with various carbon sources supplied.** Bottles on the left in all panels are uninoculated controls, and the three bottles to the right are biological triplicates. Carbon sources are cellulose only in panel A, cellulose and 2.5 g/L alkaline lignin in panel B, no carbon in panel C, and alkaline lignin alone in panel D.


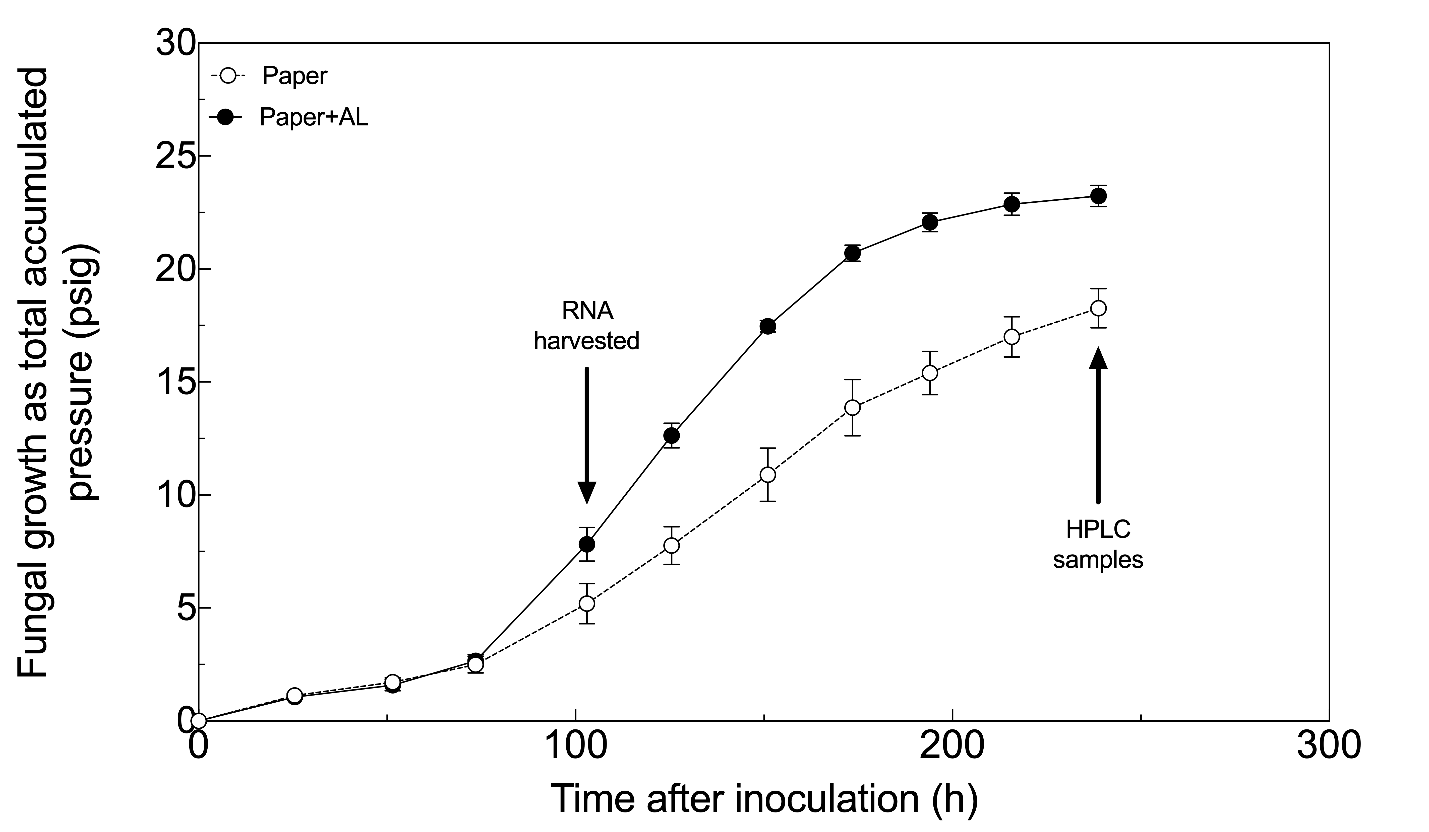


**Figure S3. A sampling scheme of *N. californiae* mRNA allows interrogation of the transcriptomic response to 0.125 g/L alkaline lignin.** These data demonstrate that cultures behaved in the same way as earlier 0.125 g/L treatments and that cultures were sampled at an appropriate juncture in the growth curve for differential expression analysis.
